# Enhancement of osteoclastogenic activity in osteolytic prostate cancer cells by physical contact with osteoblasts

**DOI:** 10.1038/sj.bjc.6606070

**Published:** 2011-01-04

**Authors:** A Shiirevnyamba, T Takahashi, H Shan, H Ogawa, S Yano, H Kanayama, K Izumi, H Uehara

**Affiliations:** 1 Department of Molecular and Environmental Pathology, The University of Tokushima Graduate School, 3-18-15, Kuramoto-cho, Tokushima 770-8503, Japan; 2 Department of Urology, Institute of Health Biosciences, The University of Tokushima Graduate School, 3-18-15, Kuramoto-cho, Tokushima 770-8503, Japan; 3 Division of Medical Oncology, Cancer Research Institute, Kanazawa University, Kanazawa, Ishikawa 920-0934, Japan

**Keywords:** physical contact, cell-to-cell interaction, prostate cancer, bone metastasis, osteoclastogenesis

## Abstract

**Background:**

The interaction between prostate cancer cells and osteoblasts is critical for the development of bone metastasis. Metastatic cancer cells may physically contact osteoblasts in the bone microenvironment; however, the biological significance of this interaction is not fully understood.

**Methods:**

Human prostate cancer cells (the osteolytic cell line PC-3 and the osteoblastic cell line MDA-PCa 2b) and human osteoblasts (hFOB1.19) were cocultured under two different conditions (bilayer and contact conditions). Differential gene expression profiles of prostate cancer cells were then investigated using microarray analysis. Differentially expressed genes were analysed using RT–PCR and western blotting, and the effect of anti-cadherin neutralising antibodies on their expression was assayed. The osteoclastogenic activity of cells grown under these different conditions was also investigated using an *in vitro* assay.

**Results:**

When PC-3 or MDA-PCa 2b cells were cocultured with hFOB1.19 cells under contact conditions, the expression of eight genes was upregulated and that of one gene was downregulated in PC-3 cells compared with gene expression in bilayer culture. No differentially expressed genes were detected in MDA-PCa 2b cells. Four of the eight upregulated genes (interleukin-1*β* (IL-1*β*), cyclooxygenase-2 (COX-2), IL-6 and the third component of complement (C3)) have already been reported to participate in osteoclastogenesis. Indeed, a cell lysate of PC-3 cells grown under contact coculture conditions significantly enhanced osteoclastogenesis *in vitro* (*P*<0.005). neutralisation of cadherin-11 with a specific antibody inhibited upregulation of COX-2 and C3 mRNA in PC-3 cells. In contrast, neutralisation of N-cadherin induced upregulation of COX-2 mRNA.

**Conclusion:**

Physical contact between osteolytic prostate cancer cells and osteoblasts may upregulate osteoclastogenesis-related gene expression in prostate cancer cells and enhance osteoclastogenesis. Additionally, cadherin-11 and N-cadherin are involved in this process. These data provide evidence supporting new therapies of prostate cancer bone metastasis that target direct cancer-cell-osteoblast cell–cell contact.

Prostate cancer continues to be the most common cancer, and the second leading cause of cancer deaths, among American men. Despite earlier diagnosis and refinements in surgery and radiation, it was estimated that 28 660 men would die from prostate cancer in the United States in 2008 (Jemal *et al*, 2008). Moreover, there has been a recent trend towards an increased incidence of prostate cancer in Asia, although the rate of prostate cancer in Asia is still much lower than that in the United States or in many European countries (Sim and Cheng, 2005). Bone metastasis of prostate cancer is the major cause of morbidity and mortality, and was detected at autopsy in up to 90% of patients who died from prostate cancer (Bubendorf *et al*, 2000). Metastasis of prostate cancer cells to bone is a multistep process including detachment of cancer cells from the primary site, travel of the cells in the blood or lymph, attachment to bone tissue and development of a tumour at the site of the bone metastasis. Prostate cancer metastases cause an osteoblastic (excessive bone forming), osteolytic (bone lysing) or mixed bone response (Charhon *et al*, 1983; Cheville *et al*, 2002; Roudier *et al*, 2003).

The interaction between prostate cancer cells and normal cells in a bone microenvironment is important for survival and proliferation of metastatic cancer cells. It has been shown that factors secreted by prostate cancer cells alter bone homoeostasis by disrupting a balance between osteoblastic and osteoclastic activity (Roudier *et al*, 2003; Logothetis and Lin, 2005; Morrissey *et al*, 2010). In turn, osteoblasts and osteoclasts secrete factors that facilitate progression of prostate cancer in bone (Lang *et al*, 1995; Yin *et al*, 2005). Once prostate cancer cells have metastasised to bone marrow, the cancer cells are suspected to interact with osteoblasts, osteoclasts and stromal cells thorough both soluble factors and physical contact. Interaction mediated by soluble factors has been studied using *in vitro* coculture systems. In those studies, the conditioned medium of cancer cells, or bilayer condition using a cell culture insert, was used for coculture (Martínez *et al*, 1996; Kido *et al*, 1997; Hullinger *et al*, 2000; Fizazi *et al*, 2003). However, there have been few studies of the mechanism by which prostate cancer cells physically contact normal cells in a bone microenvironment. Wang *et al* (2006) established a novel physical contact coculture system and showed that physical contact between prostate cancer cells and bone marrow stromal cells may act as an independent factor affecting the progression of bone metastasis. However, interaction between prostate cancer cells and other normal cells in the bone microenvironment remains unclear.

A variety of factors, such as morphogenetic proteins, adhesion molecules, chemotactic factors, cytokines and growth factors, are known to be involved in the metastasis of prostate cancer to bone (Mundy, 2002; Roodman, 2004). Adhesion molecules, in particular, may have a crucial role in the interaction between prostate cancer cells and normal cells in the bone microenvironment. N-cadherin and cadherin-11 are highly expressed in prostate cancer cells and osteoblasts, but not in normal prostate tissue. Cadherin-11 promotes bone metastasis in a mouse model and its expression increases as the tumour progresses from primary prostate cancer to metastatic disease in lymph nodes and especially in bone (Gravdal *et al*, 2007; Chu *et al*, 2008). Therefore, heterotypic interactions between cancer cells and osteoblasts mediated through homophilic adhesion molecules may have a role in bone metastasis formation and progression, although the underlying mechanisms are not fully understood. It is absolutely crucial to precisely understand these molecular mechanisms in order to develop new therapies for bone metastasis.

In this study, we compared gene expression of prostate cancer cells that produce either osteoblastic or osteolytic lesions, after coculture with osteoblasts using two different coculture systems. Our study reveals that physical contact between osteolytic prostate cancer cells and osteoblasts may upregulate the expression of specific osteoclastogenesis-related genes in prostate cancer cells and enhance osteoclastogenesis. Additionally, at least in part, this process is cadherin-11 dependent.

## Materials and methods

### Histological analysis of bone metastasis in autopsy cases

A total of 229 autopsy cases were reviewed and 37 various cancer cases with bone metastasis (including two of prostate cancer) were selected for further analysis. Autopsies had been performed at Tokushima University Hospital between 2003 and 2008.

### Cells and animals

The PC-3 human prostate cancer cell line was obtained from the Health Science Research Resources Bank (Osaka, Japan). The MDA-PCa 2b human prostate cancer cell line and hFOB1.19 immortalised human osteoblastic cell line were from the American Type Cell Culture Collection (Manassas, VA, USA). Both the PC-3 and the MDA-PCa 2b cell line were established from bone metastases, but the *in vivo* growth pattern of PC-3 cells is osteolytic (Uehara *et al*, 2003), whereas that of MDA-PCa 2b cells is osteoblastic (Fizazi *et al*, 2003). The hFOB1.19 cells were transfected with a gene coding for a temperature-sensitive mutant (tsA58) of the SV40 large T antigen and exhibit rapid growth at 33.5°C (Harris *et al*, 1995). Before coculture studies, we confirmed the mRNA expression of alkaline phosphatase and osteocalcin, markers of osteoblastic differentiation, in hFOB1.19 cells at 37°C using RT–PCR (data not shown). The PC-3 cell line was maintained in MEM supplemented with 10% fetal bovine serum (FBS), MDA-PCa 2b was maintained in BRFF-HPC1 (Athena Environmental Sciences, Baltimore, MD, USA) with 20% FBS and hFOB1.19 was maintained in phenol red-free DMEM/F12 supplemented with 10% FBS. Penicillin G (100 U ml^−1^) and streptomycin sulphate (0.1 mg ml^−1^) were added to all conditioned media. Male BALB/c mice (6–10 weeks old) and BALB/c nude mice (5 weeks old) were purchased from Crea Japan (Tokyo, Japan). Mice were housed and maintained under specific pathogen-free conditions. Experiments were performed according to the Guideline for the Care and Use of Laboratory Animals of the University of Tokushima School of Medicine, and were approved by the Animal Care and Use Committee.

### Xenograft model of osteolytic bone metastasis

PC-3 cells (5 × 10^5^ per mice) were injected into the proximal tibiae of nude mice under anaesthesia that consisted of a mixture of ketamine hydrochloride and xylazine. After 5–9 weeks, the mice were killed and the hind limbs were excised at the knee joint, fixed in 10% phosphate-buffered formaldehyde at room temperature for 24 h, and then decalcified with 10% EDTA (pH 7.4) at 4°C for 2 weeks. The tissues were then embedded in paraffin, sectioned and subjected to H&E staining and immunohistochemical staining.

### Coculture assay and fluorescence-activated cell sorting

Prostate cancer cells and hFOB1.19 cells were cocultured in contact and bilayer cocultures. Before coculture, the prostate cancer cells (PC-3 and MDA-PCa 2b cells) and hFOB1.19 cells were treated with 10 *μ*g ml^−1^ of the fluorescent dye DiOC_18_(3) or DiIC_18_(3) (Invitrogen, Carlsbad, CA, USA) for 48 h. In the contact coculture, the prostate cancer cells were mixed and cocultured with hFOB1.19 cells in serum- and phenol red-free DMEM/F12 for 48 h. The initial ratio of the cell numbers was set at 1 : 1. In the bilayer coculture, the prostate cancer cells were first seeded onto a 6-well plate (4 × 10^5^ cells per well) and a cell culture insert (PET membrane, 0.4 *μ*m pore size, Falcon, Franklin Lakes, NJ, USA) was subsequently placed on the top of each well. Equal numbers of hFOB1.19 cells were seeded onto this insert and the plate was cultured for 48 h. The medium used for this bilayer coculture was the same as that used for the contact coculture. In both types of coculture, the cells were harvested using 1 mM EDTA. Prostate cancer cells were isolated from the cocultured cell mixture using fluorescence-activated cell sorting (FACS) and the EPICS XL-MCL (Beckman Coulter, Fullerton, CA, USA). The osteoblast fraction was simultaneously isolated.

### Immunocytochemical staining

After contact coculture, some of the isolated prostate cancer cells and osteoblasts were reseeded onto MAS-coated slide glass (Matsunami Glass Ind., Osaka, Japan) and were incubated at 37°C for 24 h. These cells were fixed in 95% ethanol at room temperature for 20 min, and were then heated in 0.01 M citrate buffer (pH 6.0) for 10 min in a pressure cooker for antigen retrieval. Immunocytochemical staining was performed using the ChemMate ENVISION kit/horseradish peroxidase (DakoCytomation, Carpenteria, CA, USA). A monoclonal SV40 T antigen antibody (PAb416; Calbiochem, Darmstadt, Germany), that reacts specifically with the SV40 large T antigen but not with SV40 small T antigen, was added to the slides at a dilution of 1 : 100, and was incubated for 1 h at room temperature. After washing with PBS, each slide was treated with horseradish peroxidase-conjugated secondary antibody for 40 min. The signal was visualized using 3, 3′-diaminobenzidine, and the cells were then counterstained with hematoxylin.

### Microarray analysis

Total RNA from hFOB1.19, PC-3 and MDA-PCa 2b cells was isolated using an RNeasy Mini kit (QIAGEN, Valencia, CA, USA). Relative purity of the RNA was measured using an Agilent 2100 Bioanalyzer (Agilent Technologies, Santa Clara, CA, USA). RNA expression was analysed using a GeneChip Human Gene 1.0 ST Array (Affymetrix, Santa Clara, CA, USA). This microarray chip contains 28 869 oligonucleotide probes for known and unknown genes. First strand cDNA was synthesised from 300 ng of total RNA using GeneChip whole transcript (WT) cDNA Synthesis and Amplification kit (Affymetrix) according to the manufacturer's protocol. Complementary RNA (10 *μ*g) was input into the second-cycle cDNA reaction and then this cDNA was fragmented and end-labeled with the GeneChip WT Terminal Labelling kit (Affymetrix). Approximately 5.5 *μ*g of labelled DNA target was hybridised to the Affymetrix GeneChip Human Gene 1.0 ST Array at 45°C for 17 h on a GeneChip Hybridisation 640 (Affymetrix) according to the manufacturer's recommendation. Hybridized arrays were washed and stained on a GeneChip Fluidics Station 450, were scanned using a GeneChip Scanner 3000 7G (Affymetrix) and then CEL files were generated for each array. This analysis was supported by the Support Center for Advanced Medical Sciences, the University of Tokushima Graduate School, Institute of Health Biosciences.

### Semiquantitative RT–PCR

RNA molecules identified in the microarray analysis were subjected to semiquantitative RT–PCR analysis. Aliquots (2 *μ*g per reaction) were reverse-transcribed using SuperScript II reverse transcriptase and random hexamers (Invitrogen). This reaction was conducted at 42°C for 60 min, after which the temperature was increased to 72°C for 15 min. The total cDNA was then amplified using PCR by following a thermocycling program of 94°C for 10 min for initial denaturation, 28 cycles of 94°C for 30 s, 55°C for 1 min, 72°C for 1 min for amplification, and a final extension at 72°C for 10 min. The sequences of these primers are listed in Table 1. The RT–PCR products were separated by 1.5% agarose gel electrophoresis and were visualized using an UV transilluminator.

### ELISA and western blot analysis

After sorting, cocultured PC-3 cells were resuspended in phosphate-buffered saline containing 1 mM phenylmethylsulfonyl fluoride and were lysed using an ultrasonic homogenizer. These homogenates were then centrifuged and the supernatants were used as protein samples. The protein concentrations of the samples were quantified using the DC Protein Assay kit (Bio-Rad, Hercules, CA, USA). Aliquots (8 *μ*g protein) were subjected to ELISA assays for IL-1*β* (Human IL-1*β* ELISA kit, Invitrogen), IL-6 (Quantikine human IL-6 immunoassay kit, R&D Systems, Minneapolis, MN, USA) and C3 (AssayMax human complement C3 ELISA kit, Assaypro, St Charles, MO, USA). Aliquots (10 *μ*g protein) were also subjected to western blot analysis as described previously (Takahashi *et al*, 2008). Rabbit anti-COX-2 (Santa Cruz Biotechnology, Santa Cruz, CA, USA) and anti-actin (Sigma, St Louis, MO, USA) antibodies were used as the primary antibodies. Goat anti-rabbit IgG-horseradish peroxidase (Invitrogen) was employed as the secondary antibody. The dilutions used are as follows: anti-COX-2, 1 : 250; anti-actin, 1 : 10 000; and anti-rabbit IgG-horseradish peroxidase, 1 : 200 000. An immobilon western horseradish peroxidase substrate (Millipore, Billerica, MA, USA) was used to detect the signals.

### Immunohistochemical staining

Paraffin sections (4 *μ*m) of bone lesions from a xenograft model of osteolytic bone metastasis were deparaffinized in xylene and dehydrated through descending concentrations of ethanol. Antigen retrieval and immunostaining was performed according to the immunocytochemical staining protocol described above. A monoclonal mouse anti-COX-2 antibody (COX 229; Invitrogen) was used as the primary antibody at dilution of 1 : 50.

### *In vitro* osteoclastogenesis assay

Bone marrow was collected from male BALB/c mice by flushing the femurs and tibias with serum-free DMEM. The cells were then washed twice in the same medium, were seeded onto a 100 mm dish and were cultured in 10% FBS-containing DMEM at 37°C for 1 week. During culture, the medium was changed every three days. Adherent cells were reseeded onto a 96-well microplate (1 × 10^4^ cells per well), were preincubated at 37°C for 24 h and were then treated with each cell lysate from PC-3 cells cocultured with hFOB 1.19 cells under contact or bilayer conditions (10 *μ*g) in the presence of receptor activator for nuclear factor-*κ*B ligand (RANKL, R & D systems, Mineapolis, MN, USA) and macrophage colony-stimulating factor (M-CSF) (10 ng ml^−1^; R&D Systems) after changing to flesh complete medium. After incubation for 10 days, the cells were fixed in 5% phosphate-buffered formaldehyde at room temperature for 5 min and tartrate-resistant acid phosphatase (TRAP) staining was conducted to detect differentiated osteoclasts. The composition of the TRAP staining solution was described in our previous report (Takahashi *et al*, 2008). The number of TRAP-positive cells in more then five microscopic fields was counted at × 200 magnification.

### Neutralisation assay

PC-3 and hFOB1.19 cells were treated with DiOC_18_(3) and DiIC_18_(3), respectively. The conditions of treatment were the same as described above. Before contact coculture, the hFOB1.19 cells were treated with 40 *μ*g ml^−1^ of neutralising anti-N-cadherin (Sigma) or anti-Cadherin-11 (R&D Systems) antibody, or of the corresponding isotype IgG for 30 min at 37°C. These antibody-treated hFOB1.19 cells and PC-3 cells were mixed and cocultured in serum- and phenol red-free DMEM/F12 for 48 h. The initial ratio of cell numbers was set at 1 : 1. PC-3 cells were isolated using FACS sorting as described above. Isolated PC-3 cells were used for semiquantitative RT–PCR analyses for COX-2, IL-1*β*, IL-6 and C3 mRNA. Calculations of signal intensities were performed by using NIHimage 1.62 software (http://rsbweb.nih.gov/nih-image/).

### Statistical analyses

A two-tailed Student's *t*-test was employed for statistical analyses of the data. Significant results were determined at a *P*<0.05.

## Results

### Histological findings of direct cancer-cell-osteoblast contact in bone metastasis

**Figure 1:**
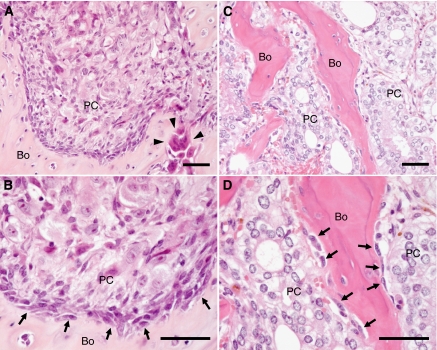
Histological findings suggesting direct prostate cancer-cell-osteoblast contact in bone metastases. The indicated tissues were fixed and analysed following H&E staining. (**A** and **B**) An osteolytic lesion produced by the growth of PC-3 human prostate cancer cells in the tibia of a nude mouse. The close proximity of PC-3 cells to osteoblasts suggesting direct cancer-cell-osteoblast contact, as well as osteolysis by osteoclasts was observed. (**C** and **D**) Representative osteoblastic bone metastases of autopsy cases of prostate cancer. The close proximity of prostate cancer cells to osteoblasts suggesting direct cancer-cell-osteoblast contact was also observed. Arrows, osteoblasts; arrow heads, osteoclasts; Bo, bone; PC, prostate cancer cells. Scale =50 *μ*m.

We first determined if cancer-cell-osteoblast cell–cell contact could be observed in bone lesions in a xenograft model of osteolytic bone metastasis. Bone surfaces facing tumours were extensively lysed by osteoclasts. However, in some areas, residual osteoblasts were observed very close to cancer cells suggesting direct cancer-cell-osteoblast contact (Figure 1A and B).

We next determined how often direct cancer-cell-osteoblast contact occurs in human tumours, by reviewing H&E-stained slides of 37 autopsy cases with bone metastases. The primary tumour sites of these cases were as follows: lung (17 cases), large intestine (4 cases), gallbladder (3 cases), pancreas, bladder and prostate (2 cases each), pleura, breast, stomach, liver, bile duct, kidney and soft tissue (1 case each). The bone metastases in the two case of prostate cancer were osteoblastic, and those in the other cases were osteolytic. The close proximity of cancer cells to osteoblasts suggesting direct cancer-cell-osteoblast contact was also observed to varying extents in all cases (Figure 1C and D).

### Immunocytochemical staining of the SV 40 large T antigen

**Figure 2:**
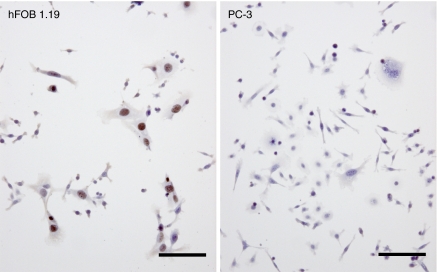
Immunohistochemical analysis of the SV40 large T antigen after sorting of cells in contact cocultures. PC-3 human prostate cancer cells (labelled with DiOC_18_(3)) were mixed and cocultured with DiIC_18_(3)-labeled human osteoblasts expressing SV40 (hFOB1.19) for 48 h. Cocultured prostate cancer cells (DiOC_18_(3)^+^/DiIC_18_(3)^−^) and hFOB1.19 cells (DiOC_18_(3)^−^/DiIC_18_(3)^+^) were then separated using flow cytometry. One thousand cells from each sorted cell population were cultured overnight on glass slides and were then immunohistochemically stained for the SV40 large T antigen. Positive staining of the SV40 large T antigen was observed in the nuclei of hFOB1.19 cells (DiOC_18_(3)^−^/DiIC_18_(3)^+^) but not in PC-3 cells (DiOC_18_(3)^+^/DiIC_18_(3)^−^). Scale =100 *μ*m.

To further analyze cancer-cell-osteoblast contact, the human prostate cancer cells (PC-3 and MDA-PCa 2b calls) and hFOB1.19 osteoblasts, which were labelled with 10 *μ*g ml^−1^ of the fluorescent dye DiOC_18_(3) and DiIC_18_(3), respectively, were cocultured in contact or in bilayer cocultures for 48 h. Following coculture in contact cocultures, the prostate cancer cells and osteoblasts were then separated by sorting for further analyses. To confirm the purity of separated cell populations (DiOC_18_(3)-labeled human prostate cancer cells and DiIC_18_(3)-labeled human osteoblasts) after sorting, the cells were immunocytochemically stained for the SV40 large T antigen, which is a marker of hFOB1.19 cells. As shown in Figure 2, the nuclei of hFOB1.19 cells (DiOC_18_(3)^−^/DiIC_18_(3)^+^) stained positive for the SV40 large T antigen, whereas no positive staining of PC-3 or MDA-PCa 2b cells (DiOC_18_(3)^+^/DiIC_18_(3)^−^) was observed (MDA-PCa 2b cells are not shown in Figure 2). Thus, prostate cancer cells and osteoblasts can be completely separated by sorting after coculture in contact cocultures.

### Gene expression analysis

We next performed cDNA microarray analysis of prostate cancer cells that had been cocultured with osteoblasts, to identify differential gene expression in prostate cancer cells because of physical contact with the osteoblasts. We compared gene expression in cocultures of both osteoblastic (MDA-PCa 2b) and osteolytic (PC-3) prostate cancer cell lines with osteoblasts under bilayer and contact culture conditions. As controls, the prostate cancer cells were cultured alone in the absence of osteoblasts. Under bilayer coculture conditions, there is no physical contact between the prostate cancer cells and the osteoblasts, but each cell line is exposed to soluble factors secreted by both cell types. In contrast, under contact coculture conditions, cancer cells and osteoblasts are both physically in contact and are exposed to soluble factors secreted by both cell types. The control prostate cancer cells cultured alone have no interaction with osteoblasts. The microarray results of the prostate cancer cells cultured alone were compared with those of prostate cancer cells from the bilayer coculture. The results of the prostate cancer cells in the bilayer coculture were further compared with those of cells from the contact coculture. A fold change in gene expression that was >3.0 or <0.33 was considered significant. There was no significant difference in gene expression between PC-3 cells cultured alone and those cocultured under bilayer conditions. This result suggested that soluble factors presents in the coculture did not affect the gene expression in PC-3 cells. In contrast, when the gene expression of PC-3 cells cultured under contact coculture conditions was compared with that of PC-3 cells cultured under bilayer coculture conditions, eight upregulated genes (3.2–5.3-fold) and one downregulated gene (0.3-fold) were identified in the contact cocultures (Table 2). Thus, the expression of these genes appeared to be specifically regulated by direct contact between PC-3 cells and osteoblasts. We then determined if these regulated genes might be related to genes that are known to be involved in cancer metastasis and bone remodelling. Four of the eight upregulated genes (IL-1*β*, COX-2, IL-6 and C3) have already been reported to participate in osteoclastogenesis (Mangham *et al*, 1993; Sato *et al*, 1993; García-Moreno *et al*, 2002; Roodman, 2004; Liu *et al*, 2005; Singh *et al*, 2007; Bussard *et al*, 2008). There was no significant difference in the gene expression of MDA-PCa 2b cells under any culture conditions.

### Validation of cDNA microarray results

**Figure 3:**
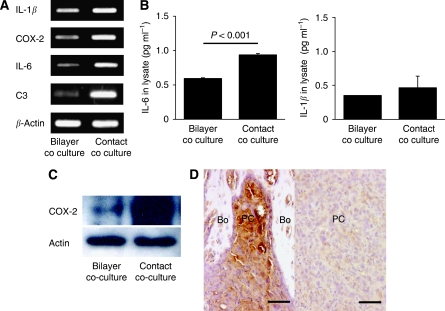
Analysis of IL-1*β*, COX-2, IL-6 and C3 expression in PC3 cells cocultured with hFOB1.19 cells. (**A**) The mRNA expression of the four osteoclastogenesis-related genes, IL-1*β*, COX-2, IL-6 and C3, in PC-3 cells following coculture under contact and bilayer conditions, was analysed using RT–PCR. Consistent with the cDNA microarray results, these genes were upregulated in contact cocultured PC-3 cells compared with bilayer cocultured cells. The primers used for RT–PCR are shown in Table 1. (**B**) Measurement of IL-6 and IL-1*β* levels in PC-3 cells using ELISA. The level of IL-6 was significantly higher (*P*<0.001) in PC-3 cells under contact coculture conditions than under bilayer coculture conditions, whereas IL-1*β* levels did not significantly differ between the two coculture conditions. ELISA was performed using a PC-3 cell lysate. (**C**) Analysis of COX-2 expression in PC-3 cells by western blotting. Expression of COX-2 was higher in PC-3 cells under contact coculture conditions than under bilayer coculture conditions. (**D**) Immunohistochemical analysis of COX-2 expression in an osteolytic bone metastasis model. Left: Strong expression of COX-2 in PC-3 cells adjacent to the bone. The close proximity of PC-3 cells to osteoblasts suggesting direct cancer-cell-osteoblast contact was observed in this area. Right: very low expression of COX-2 in PC-3 cells distant from the bone. Bo, bone; PC, prostate cancer cells. Scale=50 *μ*m.

To confirm the microarray results, the mRNA expression of the four osteoclastogenesis-related genes that were upregulated in PC-3 cells following coculture with hFOB1. A total of 19 cells under contact conditions were examined using RT–PCR. Consistent with the cDNA microarray results, upregulation of IL-1*β*, COX-2, IL-6 and C3 mRNA were detected in PC-3 cells under contact coculture conditions compared with under bilayer coculture conditions (Figure 3A).

We next determined the expression of IL-1*β*, COX-2, IL-6 and C3 in PC-3 cells at the protein level. The protein levels of IL-1*β*, IL-6 and C3 were measured using ELISA. A PC-3 cell lysate was used for ELISA, rather than culture supernatant, to eliminate the influence of exogenous IL-1*β*, IL-6 and C3 secreted by osteoblasts in the coculture. The level of IL-6 in PC-3 cells was significantly higher (*P*<0.001) under contact coculture conditions than in PC-3 cells under bilayer coculture conditions, whereas the level of IL-1*β* was not significantly different between the two coculture conditions (Figure 3B). The level of C3 in PC-3 cells was below the limits of detection under both bilayer and contact coculture conditions. Expression of COX-2 was determined by western blotting. The expression level of COX-2 was higher in PC-3 cells under contact coculture conditions than under bilayer coculture conditions (Figure 3C). Expression of COX-2 in the osteolytic bone metastasis model was also examined using immunohistochemistry. PC-3 cells formed solid tumour within the bone microenvironment in this tumour model, and strongly expressed COX-2 at the periphery of the tumour adjacent to the bone (Figure 3D, left). The close proximity of PC-3 cells to osteoblasts suggesting direct cancer-cell-osteoblast contact was locally observed in this area. In contrast, in tumour areas that were distant from the bone, COX-2 expression was very low (Figure 3D, right). These results suggest that COX-2 expression in osteolytic prostate cancer cells correlates with physical contact between the cancer cells and osteoblasts in the bone microenvironment.

### *In vitro* osteoclastogenesis

**Figure 4:**
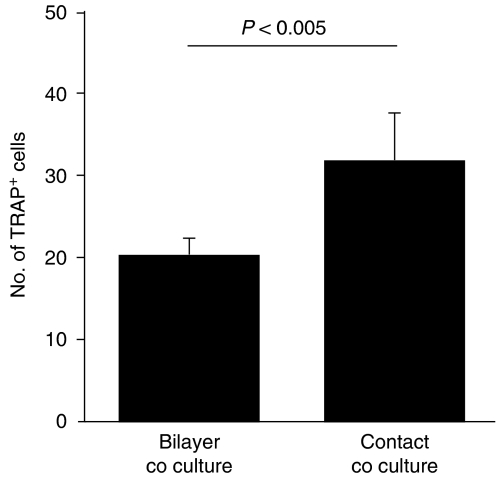
Osteoclastogenic activity of PC-3 cells cocultured with hFOB1.19 cells. The effect of addition of a cell lysate from PC-3 cells, grown under contact or bilayer coculture conditions, on osteoclastogenesis of bone marrow cells in an *in vitro* osteoclastogenesis assay, was determined by microscopic analysis of the number of tartrate-resistant acid phosphatase (TRAP) cells. The number of TRAP-positive cells in more than five microscopic fields was counted ( × 200 magnification). Osteoclastogenesis was significantly enhanced by the cell lysate of PC-3 cells grown under contact coculture conditions compared with osteoclastogenesis with a lysate of cells grown under bilayer coculture conditions (*P*<0.005). Data are representative of two separate experiments.

To examine the effect of physical contact between osteolytic cancer cells and osteoblasts on osteoclastogenic activity of cancer cells, an *in vitro* osteoclastogenesis assay was performed. In this assay, PC-3 cells that had been grown under contact or bilayer coculture conditions were lysed, and this lysate was then incubated with adherent bone marrow cells in the presence of RANKL and M-CSF for 10 days. The cells were then fixed and differentiated osteoclasts were detected by microscopic observation of TRAP staining. As shown in Figure 4, treatment with a cell lysate from PC-3 cells cocultured with hFOB1.19 cells under bilayer and contact conditions resulted in 20±3 and 31±7 TRAP-positive cells per field, respectively. The difference in number between the two culture conditions was statistically significant (*P*<0.005). These results suggest that the osteoclastogenic activity of osteolytic prostate cancer cells is enhanced by physical contact with osteoblasts.

### N-cadherin and cadherin-11 neutralisation assay

**Figure 5:**
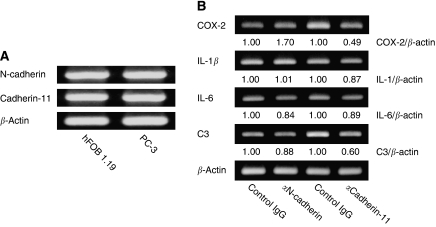
Effect of the blockade of N-cadherin- and cadherin-11-dependent cell adhesion between PC-3 and hFOB1.19 cells. (**A**) Confirmation of N-cadherin and cadherin-11 expression in both PC-3 cells and hFOB1.19 cells using RT–PCR. (**B**) Effects of N-cadherin or cadherin-11 neutralising antibodies on osteoclastogenesis-related gene expression in PC-3 cells under contact coculture conditions. The hFOB1.19 cells were pretreated with N-cadherin- or cadherin-11-neutralising antibodies for 30 min at 37°C, and then contact coculture with PC-3 cells was initiated. After 48 h, RNA was extracted from sorted PC-3 cells and was used as a template for RT–PCR to analyze the expression of the indicated osteoclastogenic genes. Signal intensities of each band were quantified by using NIHimage 1.62 software and normalised by that of *β*-actin. Data are representative of two separate experiments. The cadherin-11 neutralising antibody inhibited the upregulation of COX-2 and C3 mRNA, whereas the N-cadherin neutralising antibody further induced the upregulation of COX-2 mRNA.

We hypothesized that upregulation of osteoclastogenesis-related genes in PC-3 cells in contact cocultures may be induced by adhesion molecules that mediate interactions between PC-3 and hFOB1.19 cells. Both N-cadherin and cadherin-11 are overexpressed in osteoblasts (Marie, 2002; Mbalaviele *et al*, 2006) and prostate cancer cells (Tomita *et al*, 2000; Chu *et al*, 2008) and are considered to have an important role in bone metastasis of prostate cancer. We therefore determined if these cadherins might have a role in the upregulation of osteoclastogenesis-related genes in PC-3 cells under contact coculture conditions. For this purpose, we assayed if neutralisation of these cadherins using specific N-cadherin and cadherin-11 neutralising antibodies might inhibit the observed upregulation of these genes in PC-3 cells. We first confirmed that N-cadherin and cadherin-11 are expressed in both hFOB1.19 and PC-3 cells using RT–PCR (Figure 5A). Following pretreatment of hFOB1.19 cells with the cadherin-11 neutralising antibody, the upregulation of COX-2 and C3 mRNA that occurs in PC-3 cells under contact coculture conditions was inhibited. The ratio in level of COX-2 and C3 in anti-cadherin-11 antibody-treated cells to that in control antibody-treated cells were 0.49 and 0.60, respectively. In contrast, the upregulation of IL-1*β* and IL-6 mRNAs was not. Inversely, pretreatment with the N-cadherin-neutralising antibody further increased the upregulation of COX-2 mRNA. The ratio in level of COX-2 in anti-N-cadherin antibody-treated cells to that in control antibody-treated cells was 1.70 (Figure 5B). These results suggest that upregulation of osteoclastogenesis-related genes in PC-3 cells under contact coculture conditions is, at least in part, associated with both N-cadherin and cadherin-11.

## Discussion

In this study, we have shown that physical contact between osteolytic prostate cancer cells and osteoblasts may upregulate the expression of osteoclastogenesis-related genes in prostate cancer cells, and enhance osteoclastogenesis. Microarray analysis of genes that were only expressed under contact coculture conditions and not under bilayer conditions, were considered to be specifically induced by physical contact between prostate cancer cells and osteoblasts.

We identified eight upregulated genes and one downregulated gene in PC-3 cells that were regulated only under contact coculture conditions. Interaction between cancer cells and osteoblasts has been previously studied using *in vitro* coculture systems in which conditioned medium or cell culture inserts have been employed. The results of these types of studies only reflect the effects of soluble factors and not of direct cell–cell contact. In our study, we did not detect any genes that were differentially expressed because of coculture of PC-3 cells and osteoblasts under bilayer conditions, where the PC-3 cells are only exposed to soluble factors produced by the osteoblasts. These results suggest that the contact coculture system may be useful for understanding the molecular mechanisms of interaction between prostate cancer cells and osteoblasts and for the detection of new molecular targets for the treatment of bone metastasis. Wang *et al* previously analysed gene expression in a contact coculture of PC-3 cells and rat bone marrow stromal cells. They detected 18 genes in PC-3 cells and 10 genes in bone marrow stromal cells, expression of which was regulated only in the physical contact coculture system (Wang *et al*, 2006). However, the regulated genes that they detected did not overlap with the genes that were regulated in our coculture system, indicating that osteoblasts and bone marrow stromal cells have different roles in prostate cancer bone metastasis.

Four of the eight genes that we found to be upregulated in PC-3 cells under contact coculture conditions, that is, IL-1*β*, COX-2, IL-6 and C3 have already been reported to participate in osteoclastogenesis (Mangham *et al*, 1993; Sato *et al*, 1993; García-Moreno *et al*, 2002; Roodman, 2004; Liu *et al*, 2005; Singh *et al*, 2007; Bussard *et al*, 2008).

Cyclooxygenase-2 is an inducible prostaglandin synthesis enzyme, and is indirectly involved in bone resorption and osteoclastogenesis through prostaglandin E_2_ (PGE_2_) (Akatsu *et al*, 1989; Ohshiba *et al*, 2003). The expression of COX-2 is upregulated in many cancers. The product of COX-2, prostaglandin H_2_, is converted by PGE_2_ synthase into PGE_2_, which can stimulate cancer progression (Menter *et al*, 2010). Cyclooxygenase-2 is overexpressed in primary prostate cancer with metastatic potential and its expression is associated with death from prostate carcinoma (Richardsen *et al*, 2010). Previously we have shown that neutralisation of PGE_2_ using a soluble E-prostanoid receptor 2, inhibits the growth of PC-3 cells and tumour-induced osteoclastogenesis in a mouse model of bone metastasis (Takahashi *et al*, 2008). Thus, our microarray results, that COX-2 expression is only upregulated in osteolytic PC-3 cells and not in osteoblastic MDA-PCa 2b cells, is consistent with the evidence that COX-2 and PGE_2_ are key molecules for osteolytic bone metastasis. Furthermore, immunohistochemical analysis of an osteolytic bone metastasis model showed that COX-2 is strongly expressed in PC-3 cells that are adjacent to the bone and are locally in contact with osteoblasts, and is weakly expressed in PC-3 cells that are distant from the bone. These results suggest that the contact and bilayer coculture system may mimic the *in vivo* situation of metastatic cancer cells adjacent to and distant from the bone, respectively.

Interleukin-6 is a mediator of PGE_2_-induced suppression of osteoprotegerin production by osteoblasts and enhances osteoclast differentiation (Liu *et al*, 2005). Interleukin-6 and the IL-6 receptor have been identified in human prostate carcinoma (Siegsmund *et al*, 1994) and IL-6 acts as an autocrine and paracrine growth factor in androgen-refractory prostate cancer cells including PC-3 cells (Chung *et al*, 1999). Immunohistochemical investigation of prostate cancer metastases and xenografts showed that IL-6 is expressed in the majority of prostate cancer bone metastases and to a lesser extent in prostate cancer soft tissue metastases (Morrissey *et al*, 2010). Our data is consistent with the results of these reports.

IL-1*β* has been found to increase the formation as well as the resorptive capacity of osteoclasts in culture (Trebec-Reynolds *et al*, 2010), and can induce IL-8 production in prostate cancer cells, which promotes prostate cancer cell proliferation and migration (Tsai *et al*, 2009). Although we confirmed the cDNA microarray result of IL-1*β* mRNA upregulation using RT–PCR, the protein level of IL-1*β* in PC-3 cell lysates was not significantly different under bilayer and contact coculture conditions. The reason for this discrepancy is unknown, but it may involve posttranscriptional or posttranslational regulatory mechanisms.

C3 participates in osteoclast development by potentiating M-CSF-dependent proliferation of bone marrow cells and induction of osteoclast differentiation (Sato *et al*, 1993). The concentrations of C3 in prostatic fluid are significantly greater in patients with prostate cancer than those with benign prostate hyperplasia. However, in our study it was not possible to determine differences in the level of C3 protein between PC-3 cells under bilayer and contact coculture conditions, as the C3 protein level was below the limits of detection of ELISA analysis.

The roles of other four upregulated genes, that is, tumour necrosis factor, alpha-induced protein 6, fatty acid binding protein 4, tripartite motif-containing 31, small Cajal body-specific RNA 6 on chromosome 2 and one downregulated gene, that is, secreted protein acidic and rich in cysteine (SPARC; also known as osteonectin) on osteoclastogenesis and bone metastasis must be clarified in future studies. Especially, SPARC has been assumed to be important in human prostate cancer bone metastasis as a major bone-derived chemoattractant for prostate cancer cells (Jacob *et al*, 1999). On the other hand, downregulation of endogenous SPARC by small interference RNA accelerates prostate cancer cell-line proliferation and matrix invasiveness (Said *et al*, 2009). Participation of SPARC in osteoclastogenesis is unclear. However, SPARC stimulates the synthesis of osteoprotegerin, a physiological inhibitor of osteoclastogenesis, in human periodontal ligament cells (Fujita *et al*, 2002).

We showed that PC-3 cells that were cocultured with hFOB1.19 cells under contact conditions had significantly higher osteoclastogenic activity in an *in vitro* osteoclastogenesis assay than cells cultured under bilayer conditions. On the basis of the observed protein expression levels, it would appear that COX-2 and IL-6, rather than IL-1*β* and C3, are the major molecules that influence this enhancement of osteoclastogenesis in the contact coculture system.

Cell adhesion molecules have been suggested to have a primary role in the interactions between tumour cells and host environment (Michigami *et al*, 2000; Cowin *et al*, 2005). In particular, cadherins have been proposed to function as tumour promoter factors during cancer invasion and organ preferential metastasis (Pishvaian *et al*, 1999; Gravdal *et al*, 2007). N-cadherin and cadherin-11 are transmembrane calcium-dependent and homophilic cell–cell adhesion molecules. These molecules have been detected in prostate cancers (Tomita *et al*, 2000) and in osteoblasts (Cheng *et al*, 1998; Mbalaviele *et al*, 2006) and have been reported to be implicated in cancer invasion and human osteoblast differentiation (Kii *et al*, 2004; Chu *et al*, 2008). These finding led us to hypothesize that N-cadherin and cadherin-11 may participate in the upregulation of osteoclastogenesis-related genes that was mediated by physical contact between PC-3 and hFOB1.19 cells. Consistent with previous studies, we found that N-cadherin and cadherin-11 were both expressed in PC-3 and hFOB1.19 cells. Pre-treatment of hFOB1.19 cells with cadherin-11-neutralising antibody before initiation of the coculture inhibited the upregulation of COX-2 and C3 mRNA that was induced in PC-3 cells under contact coculture conditions. In contrast, pre-treatment with N-cadherin neutralising antibody upregulated COX-2 mRNA in PC-3 cells under contact coculture conditions. These data suggest that cadherin-11 and N-cadherin are involved in the process of osteoclastogenesis that is induced by physical contact between osteolytic prostate cancer cells and osteoblasts.

There is a great deal of evidence indicating that interaction between prostate cancer cells and osteoblasts has an important role in the survival and growth of metastatic cancer cells in a bone microenvironment (Logothetis and Lin, 2005; Morrissey *et al*, 2010). Physical contact between cancer cells and osteoblasts has been suggested to be involved in these processes. However, when we histologically analysed osteolytic tumours that were produced by intratibial injection of PC-3 cells into nude mice, the bone surfaces facing the tumours were extensively lysed by osteoclasts. Although residual osteoblasts and the close proximity of PC-3 cells to osteoblasts suggesting direct cancer-cell-osteoblast contact was observed in some areas (Figure 1A), the question remained whether this type of cell–cell contact is common in human cases of bone metastases or not. However, in all 37 autopsy cases with osteoblastic or osteolytic bone metastases that we reviewed, the close proximity of cancer cells to osteoblasts suggesting direct cancer-cell-osteoblast contact was observed to varying extents. Although we could not confirm direct cancer-cell-osteoblast contact by electron microscopy, these results suggest that direct cancer-cell-osteoblast contact is common, at least in advanced bone metastases. In future studies, it must be clarified whether metastatic cancer cells physically contact with osteoblasts in early bone metastasis, either as a single cell or as a small focus of cells.

In this study, we have shown that direct metastatic cancer-cell-osteoblast contact likely to be a common event in cases with advanced bone metastases. We further show that physical contact between osteolytic prostate cancer cells and osteoblasts may enhance the osteoclastogenic activity of prostate cancer cells by inducing upregulation of the gene expression of specific osteoclastogenesis-related genes. Additionally, this process is, at least in part, cadherin-11 dependent. These data provide evidence to support the design of new therapies of prostate cancer bone metastasis that target direct cancer-cell-osteoblast contact.

## Figures and Tables

**Table 1:** Primers for RT–PCR

**Marker**	**Forward (5′–3′)**	**Reverse (5′–3′)**	**Size (bp)**	**Reference**
COX-2	CGCAGTACAGAAAGTATC	CTCTGGATCTGGAACAC	433	Takahashi *et al*, 2008
IL-1*β*	GAGCTCGCCAGTGAAATG	TGCATCGTGCACATAAGC	336	Takahashi *et al*, 2008
IL-6	AATTCGGTACATCCTCGAC	TTCTGTGCCTGCAGCTTC	381	Takahashi *et al*, 2008
C3	CAGGCAGCATCACTAAAG	CCTTGGTCTCTTCTGATC	480	Original
N-cadherin	GACAACATTCACTGCTCA	GAACTTCATAGATACCAG	488	Original
Cadherin-11	CAAGTTACATCCACGAAG	ATCTCGGTTGTCTCTGAC	488	Original
*β*-actin	TACAATGAGCTGCGTGTGG	AGATGGGCACAGTGTGGG	226	Takahashi *et al*, 2008

**Table 2:** Up-/downregulated genes in PC-3 cells cultured under contact coculture conditions, identified by cDNA microarray analysis

**Gene bank accession no.**	**Gene name**	**Fold change^a^**
*Upregulated genes in contact coculture conditions*		
NM_007115	Tumor necrosis factor, alpha-induced protein 6	5.3
NM_000576	Interleukin 1-*β*	4.4
NM_000963	Prostaglandin-endoperoxide synthase 2 (COX2)	4.2
NM_001442	Fatty acid binding protein 4, adipocyte	4.0
NM_000600	Interleukin 6 (interferon, *β*-2)	3.7
NM_007028	Tripartite motif-containing 31	3.5
NR_003006	Small Cajal body-specific RNA 6 on chromosome 2	3.4
NM_000064	Complement component 3	3.2
		
*Downregulated genes in contact coculture conditions*		
NM_003118	Secreted protein, acidic, cysteine-rich (osteonectin)	0.3

a The microarray results of PC-3 cells in the contact coculture were compared with those of cells from the bilayer coculture. The fold change in gene expression that was >3.0 or <0.33 was considered significant.
